# Congenital heart disease diagnosed with echocardiogram in newborns with asymptomatic cardiac murmurs: a systematic review

**DOI:** 10.1186/s12887-020-02212-8

**Published:** 2020-06-30

**Authors:** Shin Ae Yoon, Woi Hyun Hong, Hwa Jin Cho

**Affiliations:** 1 Department of Pediatrics, Chungbuk National University Hospital, (28644) 1 Chungdae-ro, Seowon-gu, Cheongju-si, Chungcheongbuk-do South Korea; 2 College of Medicine, Medical Research Information Center, Chungbuk National University, (28644) 1 Chungdae-ro, Seowon-gu, Cheongju-si, Chungcheongbuk-do South Korea; 3 Department of Pathology, Inje University Busan Paik Hospital, (47392) 75 Bokji-ro, Busanjin-gu, Busan, South Korea

**Keywords:** Cardiac assessment, Asymptomatic non-syndromic cardiac murmurs, Echocardiogram, Newborns, Congenital heart disease

## Abstract

**Background:**

More than 50% of newborns with congenital heart disease (CHD) are unrecognized at birth; however, the use of echocardiogram (Echo) for diagnosing CHD in newborns with asymptomatic, non-syndromic cardiac murmurs (ANCM), has not been systematically reviewed yet. We aimed to identify the incidence of CHD diagnosed with Echo and systematically review whether Echo should be recommended in this patient group.

**Methods:**

The methodology utilized in this systematic review adhered to the Preferred Reporting Items for Systematic Reviews and Meta-Analyses framework. Using the MEDLINE, EMBASE, Web of Science, and Cochrane Library databases, we performed a systematic review of publications reporting CHD diagnosed with Echo in newborns with cardiac murmurs. The quality of the included studies was evaluated using the Study Quality Assessment Tools developed by the National Institutes of Health.

**Results:**

Of the 630 studies screened, six cohort studies, four cross-sectional studies, and two case reports were included in this review. The incidence of cardiac murmurs ranged from 0.6–8.6%. Among the 1928 newborns with ANCM, 719 (37.3%) were diagnosed with Echo as having CHD, and ventricular septal defect was the most common congenital malformation. More than 50% of the newborns showed moderate CHD necessitating outpatient cardiology follow-up, and 2.5% had severe CHD requiring immediate interventions, such as cardiac catheterization and heart surgery.

**Conclusions:**

In this systematic review, a high incidence of CHD in newborns with ANCM was detected using Echo. This indicates that the use of Echo for diagnosing CHD in healthy newborns with cardiac murmurs could be helpful in earlier detection of CHD, thereby improving clinical outcomes for newborns with severe CHD.

## Background

Congenital heart disease (CHD) is a leading cause of death in newborns [1–3]. CHD prevalence in newborns has increased globally, especially in Asia, from 0.6 per 1000 live births to 9.410 per 1000 live births, according to several recent reports [4–7]. Despite antenatal screening processes using fetal echocardiogram (Echo), more than 50% of newborns with CHD are unrecognized at birth [8–10]. As undiagnosed CHD can lead to life-threatening cardiovascular collapse and cardiac arrest, which are the primary causes of death in infants, screening newborns’ cardiac health is vital [8, 11].

Cardiac assessments, including physical examinations, have been performed for newborns after birth; however, the use of Echo for diagnosing CHD, especially in newborns with asymptomatic, non-syndromic cardiac murmurs (ANCM), has been a subject of debate [12–14]. Echo, which pediatricians use to evaluate heart function, uses sound waves to produce pictures of the movement of different parts of the heart; thus, it has become the gold standard for diagnosis of CHD in pediatric patients with cardiac murmurs and its use has expanded with advances in technology [15–18]. Almost all pediatricians agree with performing Echo for newborns with cardiorespiratory symptoms, such as cyanosis and tachypnea; chromosome abnormalities, including Down syndrome; or other non-cardiac anomalies to rule out CHD [19, 20]. The appropriate criteria for the use of Echo in children and adolescents with cardiac murmurs are well established [21–23]; however, guidelines for the use of Echo in newborns with ANCM have not yet been reported. Cardiac murmurs are commonly found in newborns and are one of the major signs of potential CHD. Moreover, pediatric cardiac murmur is the primary reason for referral and further evaluations. Therefore, CHD detection in newborns with ANCM is necessary for early diagnosis and timely treatment.

Recently, it has become easy to implement Echo in neonatal care. Previous studies reported variable CHD incidence in newborns with ANCM [24–26]; however, there is no consensus on the use of Echo for early detection of CHD in newborns with ANCM. In this systematic review, we aimed to identify the incidence of CHD diagnosed with Echo and determine whether Echo should be used in the proper management of CHD in newborns with ANCM.

## Methods

This systematic review selected CHD evaluated the usefulness of Echo in identifying CHD in newborn babies with ANCM. No systematic review has yet reported on the incidence of CHD or echocardiographic outcomes in newborns with ANCM.

### Inclusion and exclusion criteria

The inclusion criteria for published studies in this review were based on the PICOT (population/patient, intervention, comparison, outcome, and time) study design questions according to the Preferred Reporting Items for Systematic Reviews and Meta-Analyses (PRISMA) guidelines [27]. Studies were included if they met the following criteria:

1. included newborns with asymptomatic cardiac murmurs diagnosed by health professionals using echocardiogram,
2. CHD was determined based on initial screening and additional screening methods, such as physical exam, family history, and echocardiogram performed by physicians,
3. study designs included all kinds of clinical trials, such as prospective and retrospective cohort studies, observational studies, case control studies, and case reports, that explored the differential diagnosis from ANCM to CHD based on first and second screenings in full term newborns.

The following studies were excluded: animal studies; reviews, comments, editorials, conference abstracts or papers, letters, and notes; studies without a specified number of cases, that did not perform echocardiogram, or that included symptomatic patients; cases of preterm newborn babies or babies > 28 days old; and studies not in English.

### Search strategy

We searched the literature published before 4 March 2019. No restrictions were imposed in terms of publication language, time, or status. We did not restrict publication date because we aimed to maximize the number of potential studies included. Electronic database searches used both free text queries and Medical Subject Headings (MeSH) terms. Using the MEDLINE, EMBASE, Web of Science, and Cochrane Library databases, we performed a systematic review of publications reporting CHD diagnosed with Echo in newborns with cardiac murmurs. Moreover, a grey literature search strategy was adapted, as appropriate, other than the major databases in order for data retrieval as many as possible with the study objectives. The goal of the search strategy was to retrieve all relevant articles related to the topic. For management of the searched literature, Endnote version X7 (Thomson Reuters, New York, NY) was used. The details of the search strategies used are provided as an appendix to this review (Additional file 1).

### Study selection

Study selection was independently decided by two reviewers based on the predefined inclusion and exclusion criteria. Discrepancies were resolved by discussion among the reviewers. Any remaining disagreements were referred to a third author to achieve a consensus. In addition, study selection included two screening levels. The two reviewers independently screened the titles and abstracts of identified studies. Subsequently, the investigators retrieved the articles identified by the reviewers for full-text review. Thereafter, the reviewers assessed the reports to ensure that they met the inclusion criteria.

### Identification of relevant studies

**Fig. 1:**
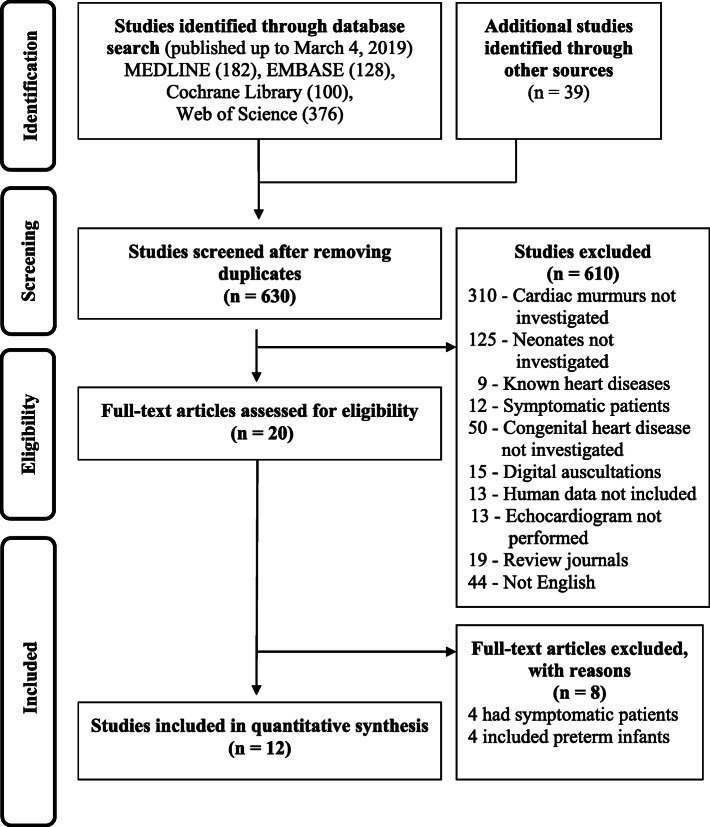
PRISMA flow diagram for identifying articles eligible for inclusion. Flow diagram of the literature search and study selection process

Figure 1 shows a flow diagram of how the relevant studies were identified. In total, 825 studies were identified by searching four databases and manually searching relevant bibliographies: In total, 786 studies were identified by searching 4 databases and manually searching relevant bibliographies as follows: 182 studies from MEDLINE, 128 from EMBASE, 100 from Cochrane Library, 376 from Web of Science, and 39 by manual searching. We excluded 156 duplicate studies and 610 studies that did not satisfy the inclusion criteria and/or met the exclusion criteria. After reviewing the full texts of the remaining 20 studies, eight studies were further excluded for the following reasons: participants included symptomatic newborns (*n* = 4) or preterm infants (n = 4) (Additional file 2). Thus, a total of 12 studies were included in the analysis [24–26, 28–36].

### Data extraction and screening methods

Two reviewers independently identified potential studies and extracted detailed information from each article according to the recommendations of the Cochrane Handbook for Systematic Reviews of Interventions Version 6 [37] and the PRISMA screening process [27] (Fig. 1).

The extracted data included indexing information about the article, such as the first author’s name, year of publication, and country where the study was performed; study design; clinical setting; total number of participants; patient demographic information, such as sex and mean age; and findings from initial screenings for CHD, additional examinations for CHD, and echocardiograms. For ANCM, the measured outcome varied among studies. The following designations of abnormal cardiac sounds were extracted from the studies: benign murmurs, possible pathologic murmurs, and pathologic murmurs.

### Quality assessment

The methodological quality of the included studies was assessed according to the Study Quality Assessment Tools developed by the National Institutes of Health (NIH) [38]. Two reviewers (S.A.Y. and H.J.C.) scored the studies according to these scales. If the ratings differed, then the authors discussed the article in an effort to reach a consensus.

## Results

### Study characteristics

Characteristics of the studies and the subjects are summarized in Table 1. Of the included studies, six were cohort studies [25, 26, 28, 29, 32, 33], four were cross-sectional studies [24, 30, 31, 34], and two were case reports [35, 36]. A total of 1928 subjects were included in the 12 studies. Clinical settings varied and included nurseries, neonatal intensive care units (NICU), and outpatient clinics.

**Table 1:** Characteristics of the studies included in the final analysis

Sources	Country	Study design	Study period	Clinical settings	Participants (n)	Sex (M/F)	Mean age (days)
Ainsworth et al. 1999, [28]	United Kingdom (Newcastle upon Tyne)	Prospective cohort study	Jan. 1995–Dec. 1996	Nursery	46	NP	NP
Rein et al. 2000, [29]	Israel (Jerusalem)	Retrospective cohort study	Jan. 1994–Dec. 1996	Nursery	170	85:85	NP
Azhar et al. 2006, [24]	Canada (Ontario/Ottawa)	Prospective cross-sectional study	Jul. 1997–Jun. 1998	NICU	75	NP	NP
Mackie et al. 2009, [30]	Canada (Quebec/ Montreal)	Prospective cross-sectional study	Oct. 2004–Jul. 2006	Outpatient	201	95:106	12
Gokmen et al. 2009, [25]	Turkey (Konya)	Prospective cross-sectional study	NP 6 months	Nursery	53	NP	NP
Mirzarahimi et al. 2011, [31]	Iran (Ardabil)	Cross-sectional study	Sept. 2006–Sept. 2007	Nursery NICU	91	NP	NP
Singh et al. 2012, [32]	United Kingdom (Birmingham)	Retrospective cohort study	Jan. 2008–Dec. 2010	Nursery	205	NP	2
Al-Ammouri et al. 2016, [33]	Jordan (Amman)	Retrospective cohort study	Aug. 2007–Jun. 2014	Nursery	309	167:142	NP
Minocha et al. 2018, [26]	United States (Florida/Miami)	Retrospective cohort study	Jan. 2013–Dec. 2016	Nursery	422	NP	NP
Fenster et al. 2018, [34]	United States (Wisconsin/Madison)	Retrospective cohort study	Jan. 2008–Dec. 2015	Nursery	354	NP	2
Ceresnak et al. 2007, [35]	United States (New York)	Case report	NP	Nursery	1	1 male	2
Paech et al. 2013, [36]	Germany (Leipzig)	Case report	NP	NICU	1	NP	NP

Abbreviations: NP, not provided; NICU, neonatal intensive care unit; M, male; F, female

### Synthesis of screening for CHD

Table 2 shows a summary of the initial and additional cardiac evaluations performed in each study. In most of the studies, the first cardiac evaluations were performed within five days of birth by pediatricians. Since 2012, screening newborns using a pulse oximeter has become standard practice [26, 32, 34, 35]. Except in one study [32], additional assessments using Echo were performed by a pediatric cardiologist during the neonatal period before discharge (an additional assessment using Echo performed in an outpatient setting was excluded [30]). Additionally, some studies used chest x-ray [24], electrocardiogram [24, 26, 30, 35], and four-limb blood pressure [26, 35] to further evaluate cardiac murmurs.

**Table 2:** Summary of the initial screening and additional assessment for cardiac murmurs in asymptomatic newborns

	Initial screening			Additional assessment	
Sources	Time	Frequency	Physicians	Time	Physicians
Ainsworth et al. 1999, [28]	≤ 48 h	NP	Neonatologist/obstetrician	Within 24 h after the murmur was heard	Pediatric cardiologist
Rein et al. 2000, [29]	≤ 5 d	Daily	Neonatologist	Median 4 h from request for echocardiogram	Pediatric cardiologist
Azhar et al. 2006, [24]	1–28 d	NP	Neonatologist	NP	Pediatric cardiologist
Mackie et al. 2009, [30]	NP	NP	NP	2–31 days	Pediatric cardiologist
Gokmen et al. 2009, [25]	NP	NP	Pediatrician	On the day of or the day after the initial screening	Pediatric cardiologist
Mirzarahimi et al. 2011, [31]	≤ 24 h	NP	Pediatrician	NP	Pediatric cardiologist
Singh et al. 2012, [32]	NP	NP	Middle grade neonatal trainee	Median age 2 days	Neonatologist
Al-Ammouri et al. 2016, [33]	≤ 48 h	NP	Pediatric resident	≤ 48 h	Pediatric cardiologist
Minocha et al. 2018, [26]	≤ 72 h	NP	NP	≤ 72 h	Pediatric cardiologist
Fenster et al. 2018, [34]	≤ 4 d	NP	NP	Median age 2 days	Pediatric cardiologist
Ceresnak et al. 2007, [35]	2nd day of life	NP	NP	On the day of or the following day after the initial screening	Pediatric cardiologist
Paech et al. 2013, [36]	NP	NP	NP	On the day of or the following day after the initial screening	NP

Abbreviations: NP, not provided

### Incidence of ANCM and CHD according to the type of cardiac murmurs

From the eight studies, the incidence of ANCM ranged from 0.6 to 8.6%, and the CHD incidence among infants with ANCM ranged from 2.6 to 74.1% (Table 3). We evaluated CHD incidence according to cardiac murmur designation in three studies [24–26]. The incidence of CHD in newborns with pathologic murmurs was higher than in those with benign or possible pathologic murmurs.

**Table 3:** Asymptomatic newborns with cardiac murmurs and congenital heart disease diagnosed with echocardiogram

Sources	Number of newborns during the study period	Number of newborns with murmurs (%)	Number of newborns with murmurs with CHD (%)
Ainsworth et al. [28]	7204	46 (0.6)	25 (54.3)
Rein et al. [29]	20,323	170 (0.8)	110 (64.7)
Azhar et al. [24]	–	75	55 (73.3)
		Innocent, 10	7 (70.0)
		Possibly pathological, 9	5 (55.6)
		Pathological, 56	43 (76.8)
Mackie et al. [30]	–	201	113 (56.2)
Gokmen et al. [25]	618	53 (8.6)	19 (35.8)
		Innocent, 41	11 (26.8)
		Pathological, 12	8 (66.7)
Mirzarahimi et al. [31]	2928	91 (3.1)	32 (35.2)
Singh et al. [32]	21,957	205 (0.9)	152 (74.1)
Al-Ammouri et al. [33]	22,215	309 (1.4)	68 (22.0)
Minocha et al. [26]	25,128	422 (1.7)	11 (2.6)
		Benign, 318	2 (0.6)
		Pathologic, 104	9 (8.7)
Fenster et al. [34]	26,573	354 (1.3)	188 (53.1)

Abbreviations: CHD, congenital heart disease

### Synthesis of echocardiographic outcomes

Of the 1928 asymptomatic newborns with cardiac murmurs who underwent Echo and were included in our study, 719 (37.3%) were diagnosed as having CHD. We classified CHD into four categories according to the anatomical region that produced the murmur: shunt lesions, right side heart lesions, left side heart lesions, and connection lesions (Table 4). Among the newborns with CHD, 56.5% had shunt lesions. The most common CHD was ventricular septal defect (VSD).

**Table 4:** Echocardiographic outcomes of newborns with cardiac murmur (*N* = 1928)^a^

Echocardiographic outcomes		n (%)	References
Shunt lesions	Ventricular septal defect	282 (14.6)	[24, 26, 28–34]
	Atrial septal defect (≥ 4 mm)	55 (2.8)	[24, 28–33]
	Ventricular septal defect + Atrial septal defect	15 (0.8)	[29, 31, 33]
	Atrioventricular septal defect	4 (0.2)	[24, 26, 32, 34]
	Significant patent ductus arteriosus	50 (2.6)	[24, 30, 34]
Right side heart lesions	Pulmonary valve anomaly (dysplasia, stenosis)	37 (1.9)	[24, 26, 28–34]
	Tetralogy of Fallot	15 (0.8)	[26, 28–30, 32–34]
	Ebstein anomaly	2 (0.1)	[29, 34]
Left side heart lesions	Cor triatriatum	1 (0.1)	[29]
	Mitral valve regurgitation	4 (0.2)	[29, 32]
	Aortic valve anomaly (bicuspid valve, stenosis)	18 (0.9)	[24, 28–30, 33, 34, 36]
	Hypoplastic left heart syndrome	3 (0.2)	[29, 33]
	Coarctation of the aorta	9 (0.5)	[28–30, 32, 34]
Connection lesions	Transposition of great arteries	6 (0.3)	[24, 29, 31]
	Partial anomalous pulmonary venous return	4 (0.2)	[26, 30, 34]
	Anomalous origin of the right pulmonary artery from the ascending aorta	1 (0.1)	[29]
Others	Coronary artery fistula	1 (0.1)	[35]
	Ventricular hypertrophy	5 (0.3)	[31]
Normal or physiologic variant		1211 (62.8)	[24–26, 28–34]
Not described		343 (17.8)	[24, 25, 32, 34]

^a^The number of diagnoses exceeds the number of patients with congenital heart disease because some patients had multiple diagnoses

### Severity of CHD in newborns with ANCM

Three studies [26, 33, 34] stratified CHD severity according to the level of interventions required (Table 5). Of the 267 newborns in the three studies, nine (3.4%) had critical CHD necessitating immediate intervention, such as heart surgery or cardiac catheterization, before hospital discharge.

**Table 5:** Severity of congenital heart disease in asymptomatic newborns with cardiac murmur

Sources	Classification of CHD severity	CHD number (%)
Al-Ammouri et al. [33]	Total CHD	68
	Critical: hypoplastic left heart syndrome, aortic stenosis	4 (5.9)
	Significant: CHD proved to be symptomatic and/or eventually required intervention during infancy or childhood	18 (26.5)
	Others	46 (67.6)
Minocha et al. [26]	Total CHD	188
	Critical: CHD requiring immediate intervention	2 (1.1)
	Major: CHD requiring intervention within 12 months of life	26 (13.8)
	Minor: CHD expected to resolve spontaneously or unlikely to require intervention	160 (85.1)
Fenster et al. [34]	Total CHD	11
	CHD triggered a change in management before hospital discharge	3 (27.3)
	CHD necessitating outpatient cardiology follow-up	8 (72.7)

Abbreviations: CHD, congenital heart disease

### Results of study quality assessment

Table 6 shows the results of each study’s quality assessment. Two reviewers rated each study according to the study design using the Study Quality Assessment Tools developed by the NIH. Scoring system was as 2 = yes; 1 = unclear or not applicable; 0 = no. A total score was graded as excellent (75–100%), good (50–75%), fair (25–50%) and poor (0–25%). Among six cohort studies, three [25, 26, 32] scored excellent while the rest [28, 29, 33] were good. Three of four cross-sectional studies [24, 30, 34] were graded as excellent and one was good. Two case reports [35, 36] scored excellent.

**Table 6:** Appraisal of study quality

Observational cohort studies (assessed with NIH tool)^a^															
	Q1	Q2	Q3	Q4	Q5	Q6	Q7	Q8	Q9	Q10	Q11	Q12	Q13	Q14	Score^b^
Ainsworth et al. [28]	2	2	2	2	1	2	2	0	0	0	2	0	2	1	18/28
Rein et al. [29]	2	2	2	2	1	2	2	0	0	2	0	0	2	1	18/28
Gokmen et al. [25]	2	0	2	2	1	2	2	2	2	2	0	2	2	1	22/28
Singh et al. [32]	2	2	2	2	1	2	2	2	2	2	0	1	2	1	23/28
Al-Ammouri et al. [33]	2	2	2	2	1	2	2	0	0	0	2	1	2	1	19/28
Minocha et al. [26]	2	2	2	2	1	2	2	2	2	1	2	1	2	1	24/28
**Cross-sectional studies** (assessed with NIH tool)															
Azhar et al. [24]	2	2	2	2	1	2	2	2	2	2	0	2	2	1	24/28
Mackie et al. [30]	2	2	2	2	1	2	2	2	2	0	2	2	2	1	24/28
Mirzarahimi et al. [31]	2	2	2	0	1	2	2	0	0	0	0	1	2	1	15/28
Fenster et al. [34]	2	2	2	2	1	2	2	1	2	1	2	1	2	1	23/28
**Case reports** (assessed with NIH tool)															
	Q1	Q2	Q3	Q4	Q5	Q6	Q7	Q8	Q9	Score					
Ceresnak et al. [35]	2	2	2	0	2	2	2	1	2	15/18					
Paech et al. [36]	2	2	2	0	2	2	2	1	2	15/18					

^a^Two assessors rated each study. In the instance of a dispute, a third assessor was involved and a consensus was reached by majority

^b^Scoring system: 2 = yes; 1 = unclear or not applicable; 0 = no.

Abbreviations: NIH, National Institutes of Health

## Discussion

This is the first systematic review to evaluate the usefulness of Echo for the diagnosis of CHD among newborns with ANCM. We found that approximately 1% of the cohort from the included studies had ANCM, and more than one third were diagnosed as having CHD using Echo. Moreover, the incidence of cardiac murmurs was influenced by the frequency and time of examination as well as the experience and skills of the physicians. Initial screening for murmurs was performed before the first discharge in most of the studies. In the study by Rein et al., cardiac auscultations were performed daily [29]. The level of expertise of the physicians who performed the initial assessments varied (i.e., pediatric residents to neonatologists). Initial cardiac evaluations for newborns consisted of cardiac auscultation, obtaining family history of CHD, and screening with a pulse oximeter, the last of which was added in 2012. Primary cardiac murmur evaluations and Echo were performed simultaneously, except in one outpatient clinic-based study [30]. Regardless of low diagnostic potential, electrocardiogram, chest x-ray, and four-limb blood pressure measurements were performed as additional assessment methods for cardiac murmurs in four studies [24, 26, 30, 35]. In this systematic review, Echo was performed by pediatric cardiologists in all but one study, in which Echo was performed by a neonatologist [32].

CHD incidence varied greatly among the studies, which could be attributable to CHD prevalence variations between different countries [4–6]. Information on antenatal ultrasound screening for CHD according to the country where the study was conducted was not available. Only one study reported that the lower CHD incidence in their study compared to previous studies could be attributed to active prenatal detection of most of the major and critical cases of CHD [26]. The incidence of CHD among full term newborns with ANCM found in this systematic review (37.3%) was much higher than the updated prevalence of CHD at birth. Among the subtypes of CHD, shunt lesions such as VSD, atrial septal defect (ASD), and patent ductus arteriosus (PDA) were most frequently observed, which is consistent with the worldwide prevalence of CHD at birth [4, 6].

With regard to the severity of CHD in newborns [39], three studies classified CHD severity as follows: mild, moderate, and severe [26, 33, 34]. Of the 267 newborns with CHD in the three studies, 3.4% were diagnosed as having severe CHD and thus required immediate interventions, such as heart surgery or interventional catheterization, before hospital discharge. In this systematic review, even though acyanotic severe heart diseases, such as large VSD, ASD, and PDA as well as critical aortic or pulmonary stenosis, were not included, 2.5% had severe CHD. The majority of the newborns had moderate CHD requiring outpatient cardiology follow-up [24, 26, 28–36].

Cardiac murmurs heard in the first few days of life are mostly associated with physiologic variants, such as peripheral pulmonary stenosis, and typically disappear within 3 to 6 months of life. Cardiac murmurs due to CHD are more easily auscultated after the decrease in pulmonary resistance that occurs after several weeks of life [40, 41]. Nevertheless, we observed a high CHD incidence in the included studies that performed initial cardiac evaluations within five days of life. Generally, pathologic murmurs that are described as louder than 2/6 in intensity, diastolic, and pansystolic and are associated with rubs, clicks, or gallops are relevant to CHD [42, 43]; however, we observed that CHD includes not only pathologic murmurs but also innocent murmurs, such as ANCM. While pathologic murmurs are associated with a higher CHD incidence, newborns with innocent murmurs showed a CHD incidence rate of 0.6–70%.

Among the included studies, only one study described an algorithm in which Echo was performed in all newborns with pathologic murmurs regardless of symptoms and in some newborns with innocent murmurs present > 48 h after birth [32]. Cardiac murmur is the most common reason for referral to a cardiologist and shows a relatively higher Echo diagnostic yield than other indications, such as palpitation or chest pain. Moreover, abnormal echocardiographic findings are most frequently observed within the first year of life among pediatric patients with cardiac murmurs [44]. Although Echo is the gold standard for the diagnosis of CHD and is easily accessible in developed countries, there is no consensus on whether to use Echo in newborns with ANCM. Nevertheless, a few physicians have performed Echo in full term newborns with ANCM [14, 34]. Although there are issues with cost-effectiveness and technical accessibility, Echo is currently widely used, even in the NICU, by neonatologists [45–48]. New approaches for the utilization of Echo in newborns with ANCM are warranted.

Early diagnosis and timely management of CHD in newborns are critical to improve clinical outcomes [5, 49]. With the increase in artificial fertilization and preterm births, CHD incidence is expected to increase in the future [50]. A thorough cardiac evaluation with careful auscultation before hospital discharge should be performed to screen for CHD. Because accuracy of the assessment of pathologic murmurs varies among physicians [24, 30, 51], a stepwise approach to evaluating cardiac murmurs is required. Considering the difficulties in distinguishing pathologic murmurs from innocent murmurs in newborns, the use of Echo in newborns with ANCM is recommended to prevent further cardiac complications. If access to Echo is limited, early referral to a pediatric cardiologist may be another option.

All studies included in this review were observational studies. A meta-analysis was impossible because of the study designs. However, this systematic review applied a comprehensive and structured search strategy to four different databases. Two reviewers independently performed quality assessment, thereby reducing selection and publication bias.

## Conclusion

Recently, Echo is being widely used for heart anatomy imaging and cardiac function evaluation in newborns with normal cardiac structure; thus, guidelines for the use of Echo in newborns with ANCM are warranted. In this systematic review, cardiac murmurs were the most common abnormal findings after physical examinations in newborns and we found that > 37% of newborns with ANCM had moderate to severe CHD diagnosed using Echo. Thus, Echo is a useful non-invasive tool for the detection of cardiac problems in newborns with cardiac murmurs and is an easy diagnostic procedure for CHD. Our study suggests that utilization of Echo in newborns with ANCM would be helpful in early CHD detection, which could in turn improve clinical outcomes.

## Supplementary information

**Additional file 1.** Detailed search strategy and returned number of articles.

**Additional file 2.** Excluded studies with reasons for exclusion.

## Data Availability

The data that support the findings of this study are available from the corresponding author (hong.medric@gmail.com) upon reasonable request.

## Abbreviations

CHD: Congenital heart disease
echo: Echocardiogram
ANCM: Asymptomatic non-syndromic cardiac murmurs
PRISMA: Preferred reporting items for systematic reviews and meta-analysis
MeSH: Medical Subject Headings
NIH: National Institutes of Health
NICU: Neonatal intensive care unit
VSD: Ventricular septal defect
ASD: Atrial septal defect
PDA: Patent ductus arteriosus
MedRIC: Medical Research Information Center
NRF: National Research Foundation of Korea
