# Supplementary material for: Congenital heart disease diagnosed with echocardiogram in newborns with asymptomatic cardiac murmurs: a systematic review
Source: BMC Pediatr. 2020 Jun 30;20:322. doi: 10.1186/s12887-020-02212-8 (PMC7325562; doi:10.1186/s12887-020-02212-8)
Supplement: Supplementary file 1 — Additional file 1. Detailed search strategy and returned number of articles. [file 12887_2020_2212_MOESM1_ESM.docx]

**Detailed search strategy and returned number of articles**

**MEDLINE**

1. (("Heart Sounds"[Mesh]) OR "Heart Murmurs"[Mesh]) OR cardiac murmur) OR Abnormal heart sound 8314
2. "Infant, Newborn, Diseases"[Mesh] OR "Infant Health"[Mesh] OR "Intensive Care Units, Neonatal"[Mesh] OR "Persistent Fetal Circulation Syndrome"[Mesh] OR "Intensive Care, Neonatal"[Mesh] OR "Infant, Newborn"[Mesh] 640261
3. (("Heart Sounds"[Mesh]) OR "Heart Murmurs"[Mesh]) OR cardiac murmur) OR Abnormal heart sound)) AND ("Infant, Newborn, Diseases"[Mesh] OR "Infant Health"[Mesh] OR "Intensive Care Units, Neonatal"[Mesh] OR "Persistent Fetal Circulation Syndrome"[Mesh] OR "Intensive Care, Neonatal"[Mesh] OR "Infant, Newborn"[Mesh]) 576
4. "Echocardiography"[Mesh] OR "Echocardiography, Four-Dimensional"[Mesh] OR "Echocardiography, Three-Dimensional"[Mesh] OR "Echocardiography, Doppler, Pulsed"[Mesh] OR "Echocardiography, Doppler, Color"[Mesh] OR "Echocardiography, Transesophageal"[Mesh] OR "Echocardiography, Doppler"[Mesh] OR "Echocardiography, Stress"[Mesh] 127429
5. 3 AND 4 182

**Cochrane Library**

1. ("murmur"):ti,ab,kw (Word variations have been searched) 94
2. heart sound 553
3. (abnormal cardiac sound):ti,ab,kw (Word variations have been searched) 13
4. (congenital heart):ti,ab,kw (Word variations have been searched) 2062
5. #1 OR #2 OR #3 OR #4 2681
6. ("echocardiogram"):ti,ab,kw (Word variations have been searched) 1190
7. electrocardiography 10923
8. #5 OR #6 OR #7 14543
9. pediatric 41533
10. MeSH descriptor: [Child] explode all trees 1125
11. MeSH descriptor: [Pediatrics] explode all trees 620
12. MeSH descriptor: [Infant] explode all trees 15136
13. MeSH descriptor: [Child, Preschool] explode all trees 378
14. MeSH descriptor: [Child, Preschool] explode all trees 378
15. MeSH descriptor: [Infant, Newborn] explode all trees 14905
16. #9 OR #10 OR #11 OR #12 OR #13 OR #14 OR #15 2357
17. #8 AND #16 in Cochrane Reviews 100

**Embase**

1. 'heart sound':ti,ab,kw OR ('cardiac murmurs':ti,ab,kw AND 'sounds':ti,ab,kw) OR 'heart murmur':ti,ab,kw 3,652
2. 'child':ti,ab,kw OR 'infant':ti,ab,kw OR 'pediatrics':ti,ab,kw OR 'preschool child':ti,ab,kw 680,995
3. #1 AND #2 259
4. 'echocardiography':ti,ab,kw OR 'echocardiogram':ti,ab,kw 194,579
5. #3 AND #4 128

**Web of Science**

1. Topic: (heart murmurs) OR Topic: (cardiac sound) ORTopic: (abnormal heart sound) OR Topic: (cardiac murmurs) 4,058
2. TOPIC: (child) *OR* TOPIC: (infant) *OR* TOPIC: (preschool) *OR* TOPIC: (pediatric*)
3. Refined by: DOCUMENT TYPES: ( ARTICLE OR MEETING ABSTRACT OR REVIEW OR PROCEEDINGS PAPER ) AND WEB OF SCIENCE CATEGORIES: ( PEDIATRICS OR SURGERY ) 385,009
4. TOPIC: (echocardio*) *OR* TOPIC: ('echocardiogram') *OR* TOPIC: ('echocardiography') 143,754
5. #7 AND #6 AND #4
6. Refined by: DOCUMENT TYPES: ( ARTICLE OR REVIEW OR PROCEEDINGS PAPER ) AND WEB OF SCIENCE CATEGORIES: ( PEDIATRICS ) 376
