# Supplementary material for: Congenital heart disease diagnosed with echocardiogram in newborns with asymptomatic cardiac murmurs: a systematic review
Source: BMC Pediatr. 2020 Jun 30;20:322. doi: 10.1186/s12887-020-02212-8 (PMC7325562; doi:10.1186/s12887-020-02212-8)
Supplement: Supplementary file 2 — Additional file 2. Excluded studies with reasons for exclusion. [file 12887_2020_2212_MOESM2_ESM.docx]

**Excluded studies with reasons for exclusion**

| **Title** | **Authors** | **Main reason for exclusion** |
| --- | --- | --- |
| *Clinical and echocardiographic evaluation of neonates with heart murmurs* | Du, Z. D., Roguin, N.  Barak, M. | Included preterm  infants |
| *Neonatal murmurs: are senior house officers good enough?* | Farrer, K. F. M.  Rennie, J. M. | Symptomatic patients |
| *A study of congenital cardiac disease in a neonatal population - the validity of echocardiography undertaken by a neonatologist* | Samson, G. R.  Kumar, S. R. | Symptomatic patients |
| *Congenital heart diseases in the newborn: from the pediatrician's request to the cardiologist's evaluation* | Rivera, I. R.  da Silva, M. A. M.  Fernandes, J. M. G. | Symptomatic patients |
| *Echocardiography and management of sick neonates in the intensive care unit* | Kadivar, M., Kiani, A.  Kocharian, A. et al. | Symptomatic patients |
| *Accuracy of Cardiac Auscultation in Asymptomatic Neonates with Heart Murmurs: Comparison Between Pediatric Trainees and Neonatologists* | Bada, H. S.; Talati, A.; John, V.; Patwardhan, A. | Included preterm  infants |
| *The pattern of congenital heart disease among neonates referred for echocardiography* | Khawahur, H. A.  Sowaiket, H. A.  Saffar, T. A. et al. | Included preterm  infants |
| *Echocardiographic Evaluation in Neonates with*  *Heart Murmurs* | Shokoufeh Ahmadipour, Azam Mohsenzadeh, Maryam Soleimaninejad | Included preterm  infants |
